# Association of cortical and subcortical microstructure with disease severity: impact on cognitive decline and language impairments in frontotemporal lobar degeneration

**DOI:** 10.1186/s13195-023-01208-7

**Published:** 2023-03-21

**Authors:** Wencai Ding, Peng Ren, Liye Yi, Yao Si, Fan Yang, Zhipeng Li, Hongbo Bao, Shi Yan, Xinyu Zhang, Siyang Li, Xia Liang, Lifen Yao, for the Frontotemporal Lobar Degeneration Neuroimaging Initiative, Howard Rosen, Bradford C. Dickerson, Kimoko Domoto-Reilly, David Knopman, Bradley F. Boeve, Adam L. Boxer, John Kornak, Bruce L. Miller, William W. Seeley, Maria-Luisa Gorno-Tempini, Scott McGinnis, Maria Luisa Mandelli

**Affiliations:** 1 Department of Neurology, The First Affiliated Hospital of Harbin Medical University, Harbin, 150001 China; 2 Laboratory for Space Environment and Physical Science, Harbin Institute of Technology, Harbin, 150001 China; 3 School of Life Science and Technology, Harbin Institute of Technology, Harbin, 150001 China; 4 Department of Neurosurgery, The Second Affiliated Hospital of Harbin Medical University, Harbin, 150001 China; 5 Department of Neurosurgery, Harbin Medical University Cancer Hospital, Harbin, 150001 China

**Keywords:** Frontotemporal lobar degeneration, Microstructure, Diffusion, Mean diffusivity, Fractional anisotropy, Biomarker

## Abstract

**Background:**

Cortical and subcortical microstructural modifications are critical to understanding the pathogenic changes in frontotemporal lobar degeneration (FTLD) subtypes. In this study, we investigated cortical and subcortical microstructure underlying cognitive and language impairments across behavioral variant of frontotemporal dementia (bvFTD), semantic variant of primary progressive aphasia (svPPA), and nonfluent variant of primary progressive aphasia (nfvPPA) subtypes.

**Methods:**

The current study characterized 170 individuals with 3 T MRI structural and diffusion-weighted imaging sequences as portion of the Frontotemporal Lobar Degeneration Neuroimaging Initiative study: 41 bvFTD, 35 nfvPPA, 34 svPPA, and 60 age-matched cognitively unimpaired controls. To determine the severity of the disease, clinical dementia rating plus national Alzheimer’s coordinating center behavior and language domains sum of boxes scores were used; other clinical measures, including the Boston naming test and verbal fluency test, were also evaluated. We computed surface-based cortical thickness and cortical and subcortical microstructural metrics using tract-based spatial statistics and explored their relationships with clinical and cognitive assessments.

**Results:**

Compared with controls, those with FTLD showed substantial cortical mean diffusivity alterations extending outside the regions with cortical thinning. Tract-based spatial statistics revealed that anomalies in subcortical white matter diffusion were widely distributed across the frontotemporal and parietal areas. Patients with bvFTD, nfvPPA, and svPPA exhibited distinct patterns of cortical and subcortical microstructural abnormalities, which appeared to correlate with disease severity, and separate dimensions of language functions.

**Conclusions:**

Our findings imply that cortical and subcortical microstructures may serve as sensitive biomarkers for the investigation of neurodegeneration-associated microstructural alterations in FTLD subtypes.

**Graphical Abstract:**

Flowchart of the study design (see materials and methods for detailed description).

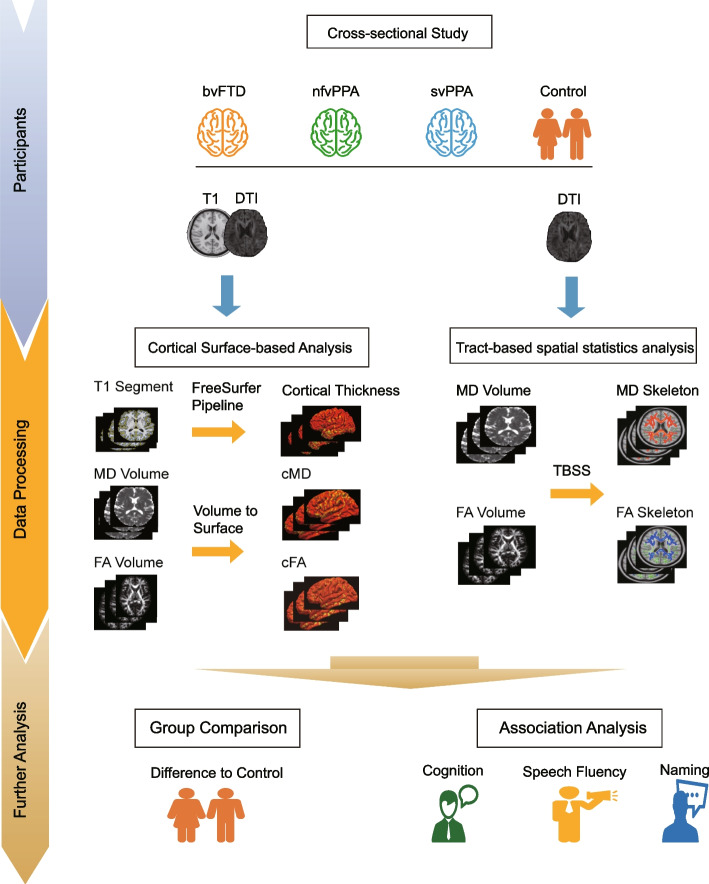

**Supplementary Information:**

The online version contains supplementary material available at 10.1186/s13195-023-01208-7.

## Introduction

Frontotemporal lobar degeneration (FTLD) denotes a neuropathological spectrum encompassing various disorders of neurodegeneration characterized by progressive deficits in executive function, behavior, or language [1, 2]. Three different prototypic FTD syndromes have been described, including behavioral variant of frontotemporal dementia (bvFTD) [3], semantic variant of primary progressive aphasia (svPPA), and nonfluent variant of primary progressive aphasia (nfvPPA) [4]. A significant proportion of patients with bvFTD exhibit highly challenging behaviors, emotions, and impulsivity and difficulty with executive function and attention [3]. Patients with nfvPPA show expressive language difficulty, characterized by hesitant speech, agrammatism, dysarthria, and telephonic errors, while cognition and behavior are preserved [4]. In those with svPPA, conceptual knowledge is gradually lost, manifested as comprehension and naming problems, with relatively good phonology and fluency and visuospatial and episodic memory [4].

Magnetic resonance imaging has emerged as a useful technology for delineating the vulnerable structures associated with FTLD [5]. Previous neuroimaging investigations have revealed that each FTLD variation is characterized by changes in gray matter macrostructure in a relatively specific regions [6–8]. In bvFTD, there was thickness atrophy in the bilateral prefrontal cortex (PFC) that spread to nearby tissues and the parietal lobe [6, 9]. In nfvPPA, supplementary motor cortex atrophy extended into the left precentral, dorsolateral, and dorsomedial prefrontal cortex [6, 10]. svPPA has been associated with cortical thinning in the orbitofrontal, temporal, and parietal cortices [6, 7]. Moreover, changes in white matter microstructures have also been found to be a major pathological characteristic in patients with variants of FTLD. Patients with bvFTD showed decreased fractional anisotropy (FA) in white matter tracts among frontal and anterior temporal regions [11–15]. nfvPPA patients showed decreased FA or increased mean diffusivity (MD) in white matter structures within the left orbitofrontal and temporal areas dorsolateral frontal and anterior temporal white matter regions [12, 15–17]. Furthermore, recent evidence has shown that in bvFTD, gray matter microstructure (mainly mean diffusivity) increases beyond areas containing the majority of the frontal and temporal cortices to posterior regions (such as the inferior parietal and occipital lobes). In primary progressive aphasia, the increase in gray matter microstructure extends beyond the dorsal anterior and anterior cingulate cortex in both hemispheres [9, 18]. Together, these imaging studies provide substantial support for extensive but relatively specific structural changes associated with each FTLD variant. However, there remain open questions regarding the white matter microstructural alterations that are associated with the different FTLD variants.

Recent advances in diffusion imaging allow for estimation of cortical microstructural organization [9, 19, 20]. Higher compartment-specific cortical MD (cMD) values suggest microstructural disruption and damage to cytomembrane and have been identified as a crucial biomarker that may precede macrostructural cortical alternations [21]. Indeed, it has been demonstrated that subtle brain structural changes that were missed by using voxel-based or surface-based morphological measurements could be detected based on cMD analysis in several neurodegenerative disorders [20, 22], even at preclinical stages [20]. Thus, cortical microstructural features may be promising for characterizing evidence of pathologic changes as well as for quantifying clinical and cognitive severity in FTLD.

To summarize, the advancement of radiomics has resulted in a variety of metrics that can now characterize various aspects of the microstructure of brain tissue based on different modalities. However, recent research has tended to explore FTLD using only a single metric or a single mode, and it remains unclear whether various metrics show the disruption of brain microstructure in FTLD differently. In addition, only a few studies have simultaneously focused on the similarities and differences among FTLD subtypes. Therefore, there is still an urgent need to use multimodal imaging data combining diverse quantification metrics simultaneously in FTLD subgroups.

In this study, we aimed to study the cortical and subcortical microstructural variations using surface-based analysis (cMD and cortical fractional anisotropy (cFA)) and TBSS (MD and FA) in a comprehensively characterized cohort of patients with varying FTLD subtypes and to evaluate their relationships with clinical measures [23–25]. Furthermore, we also explored the relationships between multimodal macrostructural/microstructural measures and the clinical scale scores representing language abilities (verbal fluency test (VFT) and Boston naming test (BNT)). As multimodal data are presented collectively, this allows us, for the first time, to investigate the differences and similarities between the different metrics. We hypothesized that cortical microstructural measurements may be more sensitive than cortical macrostructures (i.e., cortical thickness) for detecting changes associated with FTLD subtypes. We also hypothesized that cortical (cMD and cFA) and subcortical (MD and FA) microstructural analyses may provide a complementary profile of white matter pathology in FTLD subtypes.

## Materials and methods

### Study participants

Participants were recruited in the Frontotemporal Lobar Degeneration Neuroimaging Initiative (FTLDNI) database (http://4rtni-ftldni.ini.usc.edu), a multisite observational FTLD biomarker study involving 18 months of longitudinal follow-up with neuropsychological, neuroimaging, CSF, and blood examinations. Participants were enrolled at University of Mayo Clinic, Rochester (MCR), University of California, San Francisco (UCSF), and Massachusetts General Hospital (MGH).

Briefly, the inclusion criteria in this research were as follows: participants must meet criteria for bvFTD [3], nfvPPA [4], or svPPA [4] or were age-matched cognitively unimpaired (CU). All participants underwent a thorough neurological history, physical and neurological tests, structured caregiver interviews, neuroimaging, and neuropsychological testing. The cognitively normal controls were categorized by Mini-Mental State Examination (MMSE) scores ≥ 24 [26] and CDR scores = 0 [27], while performing within normal limits on all behavioral and cognitive measures [28].

The exclusion criteria for participants included a prior history of any significant neurological disease other than FTLD, including multi-infarct dementia, Parkinson’s disease, progressive supranuclear palsy, Huntington’s disease, normal pressure hydrocephalus, brain tumor, seizure disorder, alcohol or other drug abuse, significant head injury or limited language proficiency (http://4rtni-ftldni.ini.usc.edu). The Graphical Abstract depicts a systematic flowchart of the study’s design.

### MRI acquisition

MRI data (3 T) were gathered at three distinct locations for the FTLDNI database. The detailed parameters for each center’s acquisition can be obtained in in the Supplementary Table 1. Participants at three centers underwent a T1-weighted (T1w) MPRAGE acquisition with a 1-mm^3^ isotropic resolution. For consistency with most recent studies [9, 18, 19], we used four *b* = 0 and 41 directions at *b* = 1000 with an isotropic voxel size of 2.73 mm^3^.

### Surface-based analysis

T1w MRIs were processed using the cross-sectional pipelines from FreeSurfer software version 7.1.1 (https://surfer.nmr.mgh.harvard.edu). Cortical thickness was determined by computing the difference across each location of the cortical surface between representations of the white matter/gray matter and pial–CSF borders. For each individual cortical reconstruction, the white matter/gray matter boundary segmentation was visually verified slice by slice. After that, using a spherical registration procedure, each individual cortical thickness map was morphed to the FreeSurfer’s *fsaverage* surface. A Gaussian kernel with a 15-mm FWHM was applied to smooth the normalized images. Then, we processed cortical diffusion MRI using a previously established surface-based technique [20]. Briefly, diffusion MRI data were examined for significant image artifacts and excessive head motion (characterized as translation and/or rotation of more than 2 mm/2°). Diffusion-weighted imaging data that underwent quality control were denoised [29], Gibbs ringing artifacts were removed [30], eddy currents were discarded, motion was corrected between the b0 image and the diffusion-weighted acquisitions [31], and the bias field was corrected [32]. Then, diffusion tensors were fitted, and FA and MD were computed using the dwi2tensor and tensor2metric command in MRtrix3 (http://mrtrix.org). A boundary-based technique (FreeSurfer’s *bbregister*) was used to coregister the b0 scan to the segmented T1w image. Koo BB’s partial volume effect toolbox [33] was used to compute the CSF contribution in each voxel, then subtract it to estimate the net FA and MD value in the cortical gray matter. Only voxels with at least 30% gray matter, as determined by FreeSurfer’s gtmseg command [33], were considered for subsequent analyses. Then, at each vertex, FA and MD volume for each participant were registered at the midpoint between pial and white surfaces and projected to individual surfaces (FreeSurfer’s *mri_vol2surf*) to obtain individual cFA and cMD maps. Cortical surface diffusivity maps were sampled to the FreeSurfer’s fsaverage surface using spherical alignment and smoothed using a 15-mm kernel. Seventeen subjects were excluded owing to incorrect registration during preprocessing, resulting in a total of 170 participants included in the subsequent investigation.

### Tract-based spatial statistic analysis (TBSS)

The tract-based spatial analysis pipeline [34] in the FMRIB Software Library (FSL; version 6.0.4; fmrib.ox.ac.uk/fsl) was used to compare the DTI metrics between the patients with different FTLD subtypes and the cognitively unimpaired. First, all the subjects’ FA maps were nonlinearly aligned to the FMRIB-58 FA map from the Montreal Neuroimaging Institute (MNI) template space. Following the deformable registration, the mean FA skeleton was computed that represented the center of the white matter tracts common to all subjects. For MD volume, the deformation fields from FA maps were used, and the registered maps were projected onto the FA skeleton.

### Measuring disease severity, global cognition, and language disorders

All participants routinely completed the neuropsychological battery [35]. The neuropsychological evaluation included the Montreal Cognitive Assessment (MoCA) score, MMSE total score, Clinical Dementia Rating-Total score (CDR®-TOT) [28], CDR®-Sum of Boxes score (CDR®-SOB) [28], Verbal Fluency Test (VFT) [24], and the 15-item Boston naming test (BNT) [25]. To broaden the use of the Clinical Dementia Rating (CDR®) in FTLD spectrum disorders, the Behavior/Comportment/Personality (BEHAV) and Language (LANG) domains were added to the CDR®. Currently, the CDR® Dementia Staging Instrument plus National Alzheimer’s Coordinating Center Behavior and Language Domains sum of boxes (CDR® plus NACC FTLD-SB) scores are presented to measure disease severity and global cognition [23]. The VFT and BNT scores were obtained in three centers as a broad measurement of language impairment.

### Statistical analysis

Statistical analyses were conducted using the R (4.0.5) statistical program, using univariate ANOVAs to assess group differences in clinical pan and cognitive data using continuous variables. For categorical data, the chi-squared test was used. Post hoc tests using false discovery rate (FDR)-corrected were used for multiple comparisons. Cognitive and language scores that did not conform to a normal distribution were log-transformed. Statistical tests are two-sided, with significance determined by a *P* value of 0.05 or less.

We analyzed cortical thickness, cMD, and cFA between cognitively unimpaired and each of the three FTLD subgroups using a general linear model, as implemented in FreeSurfer. To connect these variations with clinical indicators of disease severity, in each of the three FTLD subgroups, a vertexwise general linear model analysis was performed using cortical thickness or cMD as dependent variable and VFT, BNT, or CDR® plus NACC FTLD-SB scores as independent variables. Age, sex, educational years, and scan site were controlled for as nuisance regressors. Corrections for multiple comparisons was implemented in FreeSurfer by using a clusterwise correction in a Monte Carlo simulation with 10,000 permutations, with the familywise error (FWE) correction set at* P* value < 0.05. Data visualization was performed using the Python package PySurfer (Pysurfer: https://pysurfer.github.io) to overlay the results onto the standard *fsaverage* surface.

TBSS was performed through a general linear model with contrasts to test for group differences between the FTLD subtypes and the cognitively unimpaired. This analysis used the TBSS framework with a nonparametric permutation test (5000 permutations) for multiple comparison correction and threshold-free cluster enhancement (TFCE) [36]. Age, sex, and site were used as nuisance covariates. The results were considered significant at *P* value < 0.05, TFCE-corrected for multiple comparisons.

### Standard protocol approvals, registrations, and patient consents

This study was approved by the institutional review boards of UCSF, MGH, and Mayo Clinic for human research. Each participant or their assigned surrogate decision maker provided informed consent.

### Data availability statement

The datasets used in this investigation are freely accessible through the Laboratory of Neuroimaging (LONI) Image Data Archive at https://ida.loni.usc.edu.

## Results

### Demographics and sample composition

For this investigation, 187 patients with satisfactory structural and diffusion-weighted MRI data were assessed. Of them, 17 subjects (9 bvFTD, 6 nfvPPA, 1 svPPA, and 1 cognitively unimpaired) were eliminated owing to processing problems. Table 1 summarizes the demographics, cognitive and clinical characteristics, and neuropsychological assessments of the subjects. There were no differences in sex, race, age at MRI acquisition, or level of education among the FTLD subgroups and cognitively unimpaired. However, FTLD subgroups showed significant differences in CDR® plus NACC FTLD-SB scores. We did not observe differences in the CDR® language subscores among the patient groups.

**Table 1:** Demographics, clinical, and neuroimaging characteristics of the participants

Characteristics	bvFTD	nfvPPA	svPPA	Cognitively unimpaired
*n*	41	35	34	60
Sex male/female,* n*	28/13^a^	17/18^a^	21/13^a^	24/36^a^
Race, white, *n* (%)	42 (100)^a^	35 (100)^a^	34 (100)^a^	60 (100)^a^
Education, years	15.1 (3.1)^a^	15.8 (2.5)^a^	15.9 (2.8)^a^	16.5 (2.0)^a^
Age at MRI, years	60.7 (6.3)^a^	68.3 (7.4)^b^	63.3 (6.3)^a^	60.1 (7.9)^e^
CDR® plus NACC FTLD-SB	9.4 (4.0)	4.3 (2.8)	5.9 (2.9)	-
CDR-BEHAV	1.7 (0.8)	0.5 (0.5)	1.1 (0.6)	-
CDR-LANG	0.9 (0.6)	1.4 (0.7)	1.0 (0.6)	-
Verbal Fluency Test (VFT)				-
Verbal fluency—D words	5.0 (4.9)^c^	5.1 (4.3)^c^	7.8 (4.6)^de^	-
Verbal fluency—animals	8.1 (6.8)^a^	8.3 (7.1)^a^	7.9 (5.3)^a^	-
Boston Naming Test (BNT)	10.9 (4.6)^c^	10.5 (5.1)^c^	4.8 (3.5)^de^	-
CDR_SB	7.0 (3.2)^ce^	2.3 (2.4)^d^	3.8 (2.1)^d^	-
Global CDR				-
0.0 = no impairment, *n (%)*	5 (12.2)	6 (17.1)	2 (5.9)	-
0.5 = questionable impairment, *n (%)*	19 (46.3)	20 (57.2)	9 (26.5)	-
1.0 = mild impairment, *n (%)*	15 (36.6)	6 (17.1)	18 (52.9)	-
2.0 = moderate impairment, *n* (%)	2 (4.9)	3 (8.6)	5 (14.7)	-
eTIV, mL	1547.8 (169.8)^a^	1473.3 (166.1)^a^	1547.6 (154.6)^a^	1474.5 (158.6)^a^

Demographics, clinical, and neuroimaging characteristics of the participants. Values reported are mean ± standard deviation

*bvFTD* behavioral variant of frontotemporal dementia, *BNT* boston naming test, *CDR® plus NACC FTLD-SB* clinical dementia rating plus national Alzheimer’s coordinating center behavior and language domains sum of boxes, *CDR-BEHAV* clinical dementia rating: behavior subscore, *CDR-LANG* clinical dementia rating: language subscore, *CDR_SB* clinical dementia rating sum of boxes, *eTIV* estimated total intracranial volume, *nfvPPA* nonfluent variant primary progressive aphasia, *svPPA* semantic variant primary progressive aphasia, *VFT* verbal fluency test

^a^Non-significant differences

^b^Different from the cognitively unimpaired (*p* < 0.05)

^c^Different from the svPPA group *(p* < 0.05)

^d^Different from the bvFTD group (*p* < 0.05)

^e^Different from the nfPPA group (*p* < 0.05)

### Neuroimaging results

#### Cortical macro- and microstructural changes in FTLD subgroups

**Fig. 1:**
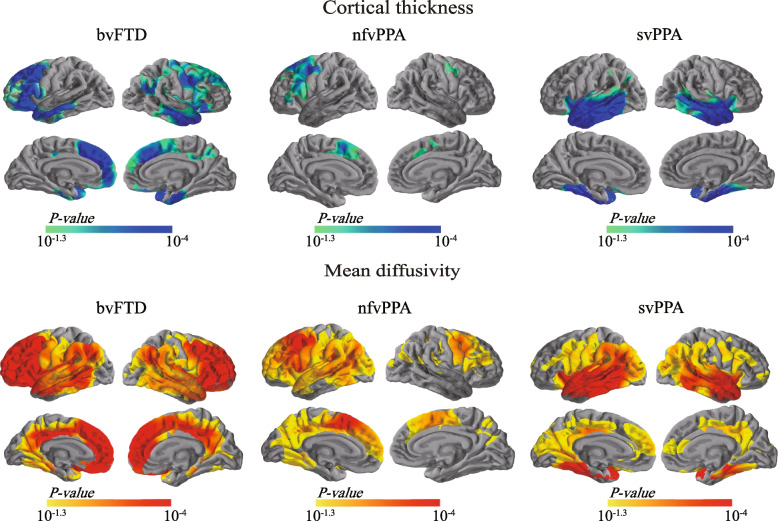
Group comparisons of cortical thickness and cortical mean diffusivity between patients with FTLD and cognitively unimpaired. Statistically significant results in cortical thickness (*top*) and cortical mean diffusivity (*bottom*) between FTLD subtypes (bvFTD, nfvPPA, and svPPA) and cognitively unimpaired individuals. Light blue–blue patches indicate thinner cortex, whereas orange–red regions indicate increased cortical mean diffusivity. Only the clusters that survived familywise error correction *P* < 0.05 are shown. bvFTD, behavioral variant of frontotemporal dementia; FTLD, frontotemporal lobar degeneration; nfvPPA, nonfluent variant primary progressive aphasia; svPPA, semantic variant primary progressive aphasia

First, we explored changes in cortical macrostructural (cortical thickness) and microstructural (cMD and cFA) in each FTLD subgroup. Figure 1 illustrates the opposite changing patterns of cortical thickness and cMD. Comparisons between the bvFTD and cognitively unimpaired subjects revealed significant cortical thinning and cMD increases in a wide range of areas, including the the anterior cingulate cortex, bilateral frontal and superior, middle temporal gyrus, insula and precuneus. In contrast, no statistically significant variations in cFA were seen between the bvFTD group and the cognitively unimpaired.

In the nfvPPA group, we found clusters of cortical thinning in the left dorsal prefrontal and premotor cortex, as well as right precentral gyrus (Fig. 1). The nfvPPA subjects presented higher cMD values in comparison to cognitively unimpaired subjects in more extensive regions, encompassing the left dorsal PFC extending to the whole frontal lobe, and left lateral temporal lobe, angular gyrus and posterior cingulate cortex (PCC)/precuneus, as well as the right medial and opercular part of the inferior frontal gyrus, and precentral gyrus. No statistical differences in cFA were observed between the nfvPPA and the cognitively unimpaired.

In the svPPA, we found local clusters of cortical thinning in bilateral temporal lobe (Fig. 1). Notably, we found significant cMD increases in the bilateral medial and lateral temporal lobes, which extended to the angular gyrus and the precuneus/posterior cingulate cortex (Fig. 1). No statistical differences in cFA were observed between the svPPA and the cognitively unimpaired.

#### TBSS alterations in FTLD subgroups

**Fig. 2:**
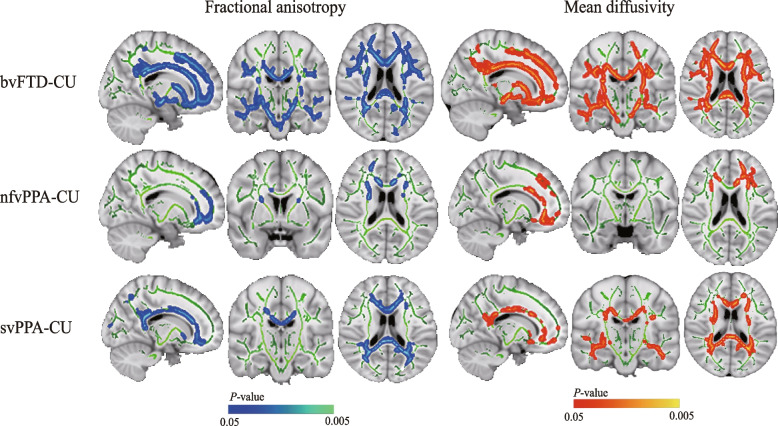
Group comparisons of TBSS between patients with FTLD and cognitively unimpaired. *Left*: Regions where fractional anisotropy values in the FTLD subtypes are considerably lower than in cognitively unimpaired within the white matter skeleton (green) are reported on a light blue–blue scale (0.05 < *P* < 0.005). *Right*: Regions where mean diffusivity values in the FTLD subtype are considerably higher than in cognitively unimpaired within the white matter skeleton (green) are reported on a red–orange scale (0.05 < *P* < 0.005). The results have been overlaid on a skull-stripped MNI152 template. bvFTD, behavioral variant of frontotemporal dementia; CU, cognitively unimpaired nfvPPA, nonfluent variant primary progressive aphasia; svPPA, semantic variant primary progressive aphasia; TBSS, tract-based spatial statistics

In comparison to the cognitively unimpaired, the bvFTD patients showed higher MD and lower FA mainly in the inferior and superior longitudinal fasciculus, CST, inferior fronto-occipital fasciculus, cingulum, forceps minor, and forceps major bilaterally (Fig. 2). The nfvPPA patients exhibited reduced FA and increased MD mainly in small clusters, including the forceps minor, bilateral anterior thalamic radiation, uncinate fasciculus, and anterior cingulum bundle (Fig. 2). The svPPA patients showed reduced FA most evidently in the cingulum, forceps minor, and forceps major, and a broader range of regions with increased MD were found (Fig. 2).

### Associations between cortical macro- and microstructures and clinical assessments

#### Correlations with CDR® plus NACC FTLD-SB scores

**Fig. 3:**
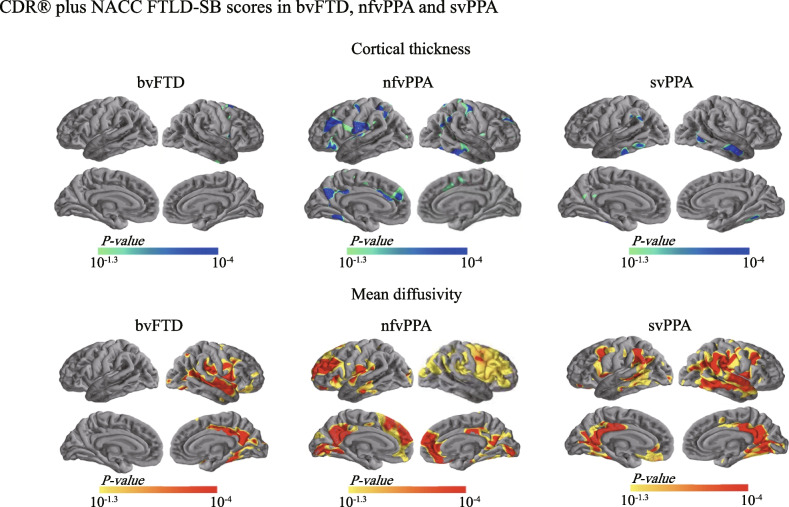
Relationship between cortical thickness and cortical mean diffusivity with CDR® plus NACC FTLD-SB scores. Correlations of cortical thickness (*top*) and cortical mean diffusivity (*bottom*) with the CDR® plus NACC FTLD-SB scores in each FTLD subgroup. Small regions of cortical thinning associated with higher CDR® plus NACC FTLD-SB scores (light blue–blue) were found in the bvFTD, nfvPPA, and svPPA patients, whereas extensive areas of increased cortical mean diffusivity related to increases in CDR® plus NACC FTLD-SB scores (orange–red) were found in each subgroup. Only the clusters that survived familywise error correction *P* < 0.05 are shown. bvFTD, behavioral variant of frontotemporal dementia; CDR® plus NACC FTLD-SB, clinical dementia rating plus national Alzheimer’s coordinating center behavior and language domains sum of boxes; FTLD, frontotemporal lobar degeneration; nfvPPA, nonfluent variant primary progressive aphasia; svPPA, semantic variant primary progressive aphasia

The scores can range from 0 to 24, with higher scores denoting more disability. First, we assessed the capability of cMD and cortical thickness to represent disease severity in each FTLD subgroup. In the bvFTD patients, while cortical thickness in only a small cluster located in the right dorsomedial premotor cortex showed a significant opposite relationship with the scores (Fig. 3), cMD in a much wider range of regions, including the right insula, supramarginal gyrus, and PCC/precuneus, exhibited positive correlations with the scores (Fig. 3). In the nfvPPA patients, the scores were found to be negatively associated with cortical thickness but positively associated with cMD in clusters in the ventrolateral and medial PFC (vlPFC and mPFC), precentral gyrus, inferior temporal cortex, angular gyrus, and PCC/precuneus (Fig. 3). In the svPPA patients, we observed an opposite relationship between cortical thickness in a limited region in the bilateral temporal cortices and the scores (Fig. 3). In contrast, we found larger clusters of significant positive correlations between the scores and cMD in the bilateral temporal cortices, angular gyri, supramarginal gyri, PCC/precuneus, and fusiform gyri (Fig. 3).

#### Correlations with VFT scores

**Fig. 4:**
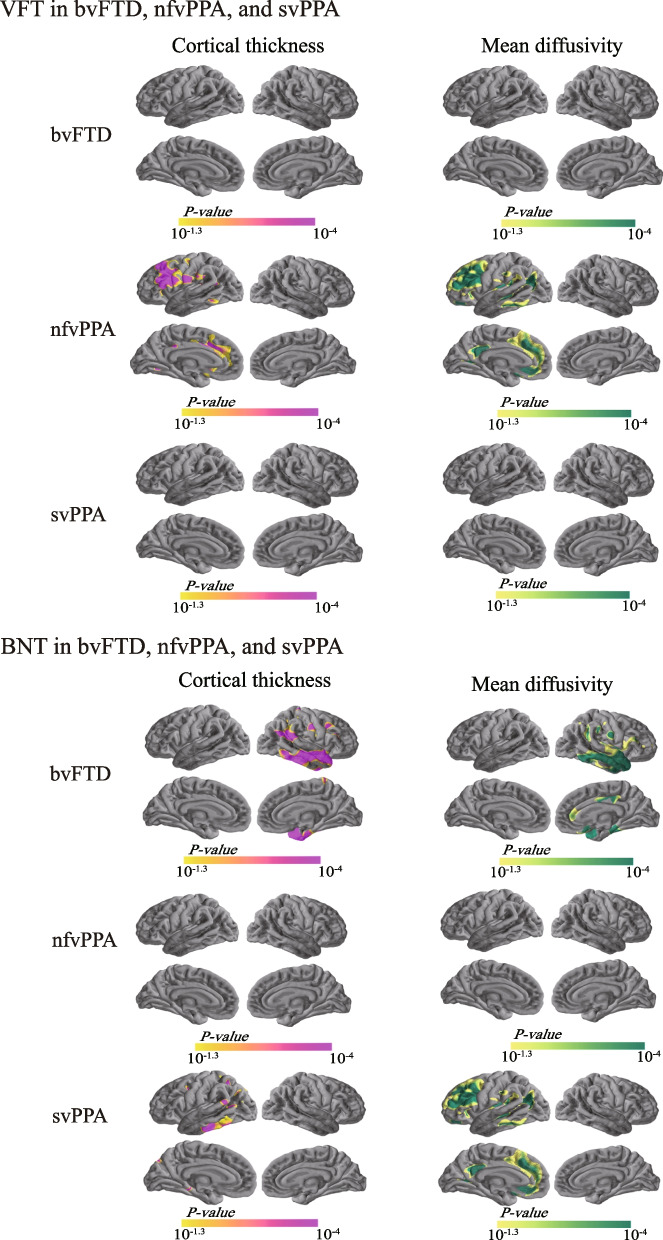
Correlations of cortical thickness and cortical mean diffusivity with VFT and BNT scores. Correlations of cortical thickness (*left*) and cortical mean diffusivity (*right*) with VFT and BNT scores in each FTLD subgroup. VFT and BNT values positively correlated with cortical thickness (orange–purple) and negatively correlated with cortical mean diffusivity (yellow–green). Only the clusters that survived familywise error correction P < 0.05 are shown. bvFTD, behavioral variant of frontotemporal dementia; BNT, boston naming test; FTLD, frontotemporal lobar degeneration; nfvPPA, nonfluent variant primary progressive aphasia; svPPA, semantic variant primary progressive aphasia; VFT, verbal fluency test

We assessed the relationships between cortical degeneration and VFT scores, a cognitive measure of verbal fluency, to explore whether their relationship would be specific to the nfvPPA patients who are characterized as showing expressive language difficulties. We found that in the nfvPPA subjects, VFT scores were positively correlated with cortical thickness in regions of the left middle lateral PFC and dorsal ACC (Fig. 4) and negatively correlated with cMD in a similar set of regions, as well as in the left mPFC, rostral ACC and PCC/precuneus (Fig. 4). We found no significant relationships between VFT scores and cortical thickness or cMD in the bvFTD or svPPA patients (Fig. 4).

#### Correlations with BNT scores

We next assessed whether cortical thickness and cMD correlated with BNT scores (ranging from 0 to 18), a cognitive measure of naming ability. Since the svPPA patients, among the three FTLD variants, are commonly associated with gradual loss of conceptual knowledge that manifests as comprehension and naming problems, we hypothesized that BNT scores might correlate with cortical degenerations in the svPPA subgroup, specifically in conceptual knowledge-related brain regions. As expected, our results showed that in the svPPA participants, BNT scores were positively associated with cortical thickness but were negatively associated with cMD in regions distributed in the left inferior and posterior temporal gyri (Fig. 4). Interestingly, we also observed that in the bvFTD subgroup, BNT scores were positively correlated with cortical thickness in the medial and lateral temporal cortices, supramarginal gyrus, and angular gyrus in the right hemisphere (Fig. 4). Negative correlations were observed between BNT scores and cMD in the above areas, as well as regions in the right anterior and posterior cingulate cortices (Fig. 4). We did not observe any significant relationship between BNT scores and cortical thickness or cMD in the nfvPPA subjects (Fig. 4).

### Associations between subcortical microstructure and clinical assessments

#### Correlations with CDR® plus NACC FTLD-SB scores

**Fig. 5:**
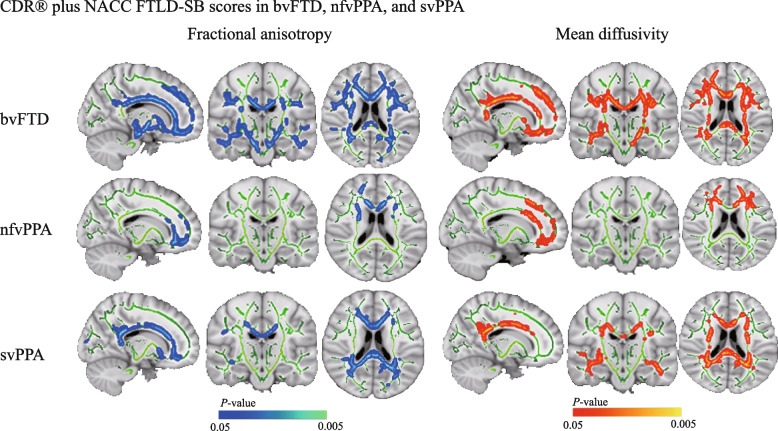
Relationships between subcortical fractional anisotropy and subcortical mean diffusivity with the CDR® plus NACC FTLD-SB scores. Correlations of subcortical fractional anisotropy (*left*) and subcortical (*right*) with CDR® plus NACC FTLD-SB scores in each FTLD subgroup. Regions of lower fractional anisotropy associated with higher CDR® plus NACC FTLD-SB scores (blue-light blue) were found in the bvFTD, nfvPPA, and svPPA patients, whereas regions of increased mean diffusivity related to increases in CDR® plus NACC FTLD-SB scores (red–orange) were found in each subgroup. The results have been overlaid on a skull-stripped MNI152 template. bvFTD, behavioral variant of frontotemporal dementia; BNT, boston naming test; CDR® plus NACC FTLD-SB, clinical dementia rating plus national Alzheimer’s coordinating center behavior and language domains sum of boxes; FTLD, frontotemporal lobar degeneration; nfvPPA, nonfluent variant primary progressive aphasia; svPPA, semantic variant primary progressive aphasia; VFT, verbal fluency test

We revealed that the scores negatively associated with FA and positively associated with MD in extensive tracts in the bvFTD group (Fig. 5). In the nfvPPA subgroup, higher scores were associated with more severe white matter damage, specifically with lower FA and higher MD in multiple frontal areas, involving the anterior thalamic radiation, forceps minor, and anterior cingulum bundle (Fig. 5), and with the forceps major, forceps minor, and cingulum in the svPPA subgroup (Fig. 5).

#### Correlations with VFT scores

**Fig. 6:**
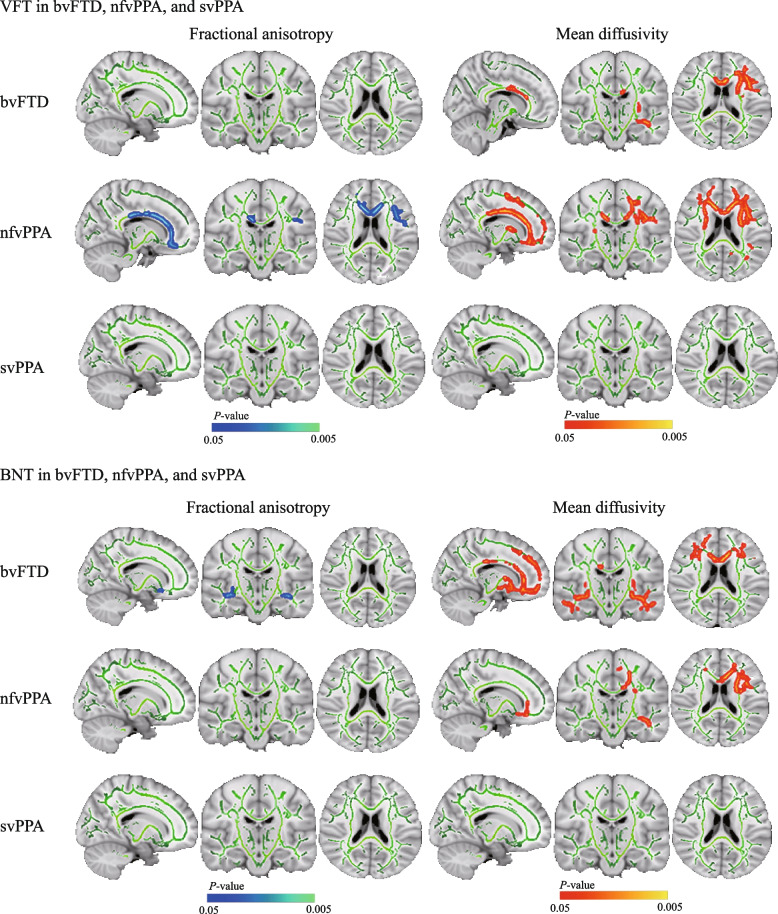
Correlations of subcortical fractional anisotropy and subcortical mean diffusivity with VFT and BNT scores. Correlations of subcortical fractional anisotropy (*left*) and subcortical mean diffusivity (*right*) with VFT and BNT scores in each FTLD subgroup. *Top*: Regions of lower fractional anisotropy associated with higher VFT scores (blue-light blue) were found in the nfvPPA patients, whereas regions of increases in mean diffusivity related to increases in VFT scores (red–orange) were found in the bvFTD and nfvPPA patients. *Bottom*: Regions of lower fractional anisotropy associated with higher BNT scores (bluelight–blue) were found in the bvFTD patients, whereas regions of increases in mean diffusivity related to increases in VFT scores (red–orange) were found in the bvFTD and nfvPPA patients. The results have been overlaid on a skull-stripped MNI152 template. bvFTD, behavioral variant of frontotemporal dementia; BNT, boston naming test; FTLD, frontotemporal lobar degeneration; nfvPPA, nonfluent variant primary progressive aphasia; svPPA, semantic variant primary progressive aphasia; VFT, verbal fluency test

In the bvFTD participants, we found a significant correlation between higher VFT scores and lower MD in the forceps minor, arcuate fasciculus, fornix, and cingulum dorsal, while no significant correlations were found with FA (Fig. 6). In the nfvPPA participants, decreased VFT scores were found to be associated with lower FA in the left cingulum, forceps minor, and arcuate fasciculus. In contrast, a larger extent of clusters in the frontal lobe was found to show significant positive correlations between VFT scores and MD (Fig. 6). The svPPA subgroup showed no correlations with VFT scores (Fig. 6).

#### Correlations with BNT scores

In the bvFTD subgroup, BNT scores positively correlated with FA in the arcuate fasciculus in both hemispheres and negatively correlated with MD in these brain regions and extended to the anterior thalamic radiation, fornix, cingulum, and orbital frontal white matter skeleton (Fig. 6). In the nfvPPA subgroup, increased MD was observed in the left corona radiata, body and genu of the anterior and posterior limbs of the internal capsule, corpus callosum, fronto-occipital fasciculus, and inferior longitudinal fasciculus with decreasing BNT scores; no significant correlation was observed between FA and BNT scores (Fig. 6). The svPPA subgroup showed no correlation between BNT scores and FA or MD (Fig. 6).

## Discussion

In this study, we analyzed the cortical macro- and microstructural changes, as well as subcortical white matter tract-based spatial statistics across FTLD subtypes. The main findings of this study are as follows: (i) patients with FTLD showed substantial cMD changes in regions that lacked cortical thinning, showing that the cMD measure may be capable of detecting microstructural changes before gray matter loss; (ii) bvFTD, nfvPPA, and svPPA patients exhibited distinct patterns of cMD increases and cortical thickness decreases; (iii) correlation analyses revealed significant relationships between cMD and disease severity across locations with significant cMD changes; (iv) significant and distinct disruptions of subcortical white matter microstructures were observed in different FTLD subtypes; (v) crucial components of these regions strongly correlated with disease severity and with language functions in specific FTLD subgroups.

We found that all three subtypes of FTLD patients showed more significant and extensive changes in cMD than in cortical thickness. Specifically, cMD increases were not restricted to regions of cortical thinning but also included areas that are prone to FTLD. These results suggested that cMD might be more sensitive than cortical thinning in identifying cortical abnormalities in all three types of FTLD. Notably, this observation was further reinforced by our correlation analysis with clinical measures of cognitive functioning (VFT and BNT scores) and disease severity (CDR® plus NACC FTLD-SB scores). These are consistent with the results described in a previous study [18]. Both clinical measures of cognitive function and disease severity showed better correlations with cMD than with cortical thickness. Recent studies reported increased cMD as a crucial and noninvasive biomarker for cortical surveillance of neurodegeneration-related microstructural alterations in possible and probable bvFTD [9], ALS-FTD continuum [22], Alzheimer’s disease (AD) continuum [20], autosomal dominant Alzheimer’s disease [37], and even normal aging adults [38]. Higher cMD is thought to predict macroscopic cortical alterations by reflecting microstructural disarray and rupture of cellular membranes [21]. It has been proposed that microstructural changes in cortical structure precede macrostructural changes in cortical structure [9, 22, 38–40]. Our research expands on these findings by using a large sample of individuals with three unique FTLD subgroups.

Subcortical white matter tract abnormalities in FTLD are relatively understudied compared with primary neurodegenerative diseases like Alzheimer’s disease. Although the precise neurological correlations remain elusive, several studies have suggested a key association of white matter abnormalities in neurodegeneration [41, 42]. These studies have demonstrated that subcortical MD and FA are measures of anisotropy of free water diffusion in white matter tracts, with the axon and myelin being destroyed, adding to the anisotropy [41, 42]. The patients with bvFTD showed a widespread pattern of MD and FA abnormalities affecting most of the white matter bilaterally. The alterations in this group were significantly more diffusely distributed across the frontotemporal and posterior regions than those in the other two groups. These widespread anomalies are consistent with previous reports [11, 12] and support the notion of FTLD network disruption impacting widely distributed frontotemporal networks rather than localized areas of damage [43, 44]. The lateral and medial parietal lobes are typically impacted later in the illness course in classic frontotemporal instances [45].

Interestingly, we observed that distinct spatial distributions of brain microstructure are related to separate dimensions of language functions in different FTLD subtypes. In the svPPA subgroup, a specific relationship was found between BNT scores and cMD mainly in the left posterior temporal cortices, which aligns well with the specific deficits in conceptual knowledge usually seen in patients with svPPA. The decline in conceptual knowledge found with svPPA occurs in the setting of substantial atrophy of the anterior temporal lobe, giving rise to the influential idea of the anterior temporal lobe as a “semantic center” [46]. Snowden JS. et al. [47] identified significant inverse relationships between atrophy in temporal regions, especially the fusiform gyrus and temporal pole, and naming scores. This is partially consistent with our present results. In contrast to the left-predominant pattern of atrophy (vs. a right-predominant pattern) associated with svPPA. In bvFTD, we found that BNT scores negatively correlated with cMD in the temporoparietal cortices and anterior and posterior cingulate cortices in the right hemisphere. There are several explanations for this. First, although bvFTD is characterized by localized and significant bilateral frontal atrophy, some analyses have shown that the right hemisphere is more involved than the left [48]. Second, fMRI studies have revealed evidence that semantic processing may be bilateral and that concrete word semantics may engage right hemisphere regions [49, 50]. Third, in the case of language production, the role of language counterpart regions in the right hemisphere and the orientation of naming performance is still being contested, particularly whether the right frontal cortices, including homologs of Broca’s area, play a positive or negative role in recovery [43, 51]. It has been established that outcomes related to language output in chronic aphasic individuals is connected with anatomical changes in language homolog areas in the right hemisphere [51]. Finally, the interpretation of naming difficulties in bvFTD is difficult [47]. Due to the clinical heterogeneity in FTD, abnormal brain regions impacting naming skill may not apply equally to all individuals [44]. Despite the fact that the between-group differences analysis revealed that bvFTD was widely distributed in cortical thickness and cMD impairment bilaterally, the BNT correlation analysis results revealed that right hemisphere cMD was more predictive of patients’ cognitive impairment of language. This could be due to the severity of the damage or the progression of the left and right cMD. The differences in right-sided cMD were found to be smaller than the differences in outcome between groups. This may indicate greater individual-level variation with stronger correlations with cognitive scores.

In the nfvPPA subgroup, VFT performance was specifically correlated with cMD in the left frontal–temporal cortex, as well as subcortical white matter microstructures near these regions. Impairments in verbal fluency are commonly seen in nfvPPA patients, which has been considered to be a dispersed cognitive capacity spanning several domains and reliant on the integrity of multiple white matter tracts [46, 47]. Previous studies have demonstrated that the main characteristics of nfvPPA, including agrammatism and apraxia of speech, are related to destruction of the left posterior fronto-insular, striatum and premotor regions, in addition to the supplementary motor area [52, 53]. Neuropsychological and functional MRI investigations have shown that posterior temporal areas play a role in syntactic processing, including comprehension and production [54]. This prior evidence is consistent with our findings.

Most previously mentioned studies using the DTI technique in FTLD patients have solely focused on cortical or white matter integrity. We show, for the first time in three FTLD subtypes using multimodal approach, that cortical and subcortical mean diffusivity correlated strongly and differentially with VFT and BNT scores. While cortical and subcortical MD metrics exhibited similar sensitivity when correlating with VFT in nfvPPA patients, and with BNT in bvFTD patients, cMD appears to be more sensitive when correlating with BNT in svPPA patients. Moreover, TBSS-MD seems to be of higher sensitivity in correlating with VFT in bvFTD patients and with BNT in nfvPPA patients. By comparing the outcomes from different modalities, our findings suggest that the sensitivity of microstructure assessments varies in FTLD subgroups and clinical scores (VFT and BNT scores), which may help to elucidate the varying roles of cortical and WM diffusivity in contributing to specific cognitive impairments in different subgroups of FTLD.

This study also has several limitations. First, our study was cross-sectional, so it was not possible to evaluate changes in white matter disease over time. Second, although surface-based pipelines were used to avoid CSF contamination, there may still be a partial volume effect resulting from low-resolution diffusion-weighted imaging of the cortical space. Third, cortical and subcortical microstructures are indirect assessments of brain parenchyma physical parameters such as white matter axon density, caliber, and myelination [55]. The pathogenic origins of aberrant diffusion remain unknown. Future research could take advantage of advanced neuroimaging technologies to identify biophysically significant traits that are more particular to axonal or myelin deterioration.

## Conclusions

In summary, this study provides evidence suggesting the existence of extensive pathological involvement in the three subtypes of FTLD and suggests that cMD may be more sensitive than cortical thinning for identifying cortical alterations responsible for cognitive and behavioral impairments in FTLD. Our findings also indicate that cortical and subcortical microstructures are related to cognition and disease severity in FTLD and could serve as a valuable tool for monitoring disease severity.

## Supplementary Information

**Additional file 1: Supplementary Table 1.** Structural T1 and diffusion weighted image acquisition protocols.

## Data Availability

The datasets used in this investigation are freely accessible through the Laboratory of Neuroimaging (LONI) Image Data Archive at https://ida.loni.usc.edu.

## Abbreviations

AD: Alzheimer’s disease
ANOVA: Analysis of variance
bvFTD: Behavioral variant of frontotemporal dementia
BNT: Boston naming test
CDR® plus NACC FTLD-SB: Clinical dementia rating plus national Alzheimer’s coordinating center behavior and language domains sum of boxes
cFA: Cortical fractional anisotropy
cMD: Cortical mean diffusivity
CU: Cognitively unimpaired
FA: Fractional anisotropy
FTLD: Frontotemporal lobar degeneration
MMSE: Mini-Mental State Examination
MD: Mean diffusivity
nfvPPA: Nonfluent variant primary progressive aphasia
TBSS: Tract-based spatial statistic
svPPA: Semantic variant primary progressive aphasia
VFT: Verbal fluency test
