# Supplementary material for: Association of cortical and subcortical microstructure with disease severity: impact on cognitive decline and language impairments in frontotemporal lobar degeneration
Source: Alzheimers Res Ther. 2023 Mar 21;15:58. doi: 10.1186/s13195-023-01208-7 (PMC10029187; doi:10.1186/s13195-023-01208-7)
Supplement: Supplementary file 1 — Additional file 1: Supplementary Table 1. Structural T1 and diffusion weighted image acquisition protocols. [file 13195_2023_1208_MOESM1_ESM.docx]

**Supplementary table 1. Structural T1 and** **diffusion weighted image acquisition protocols.**

|  | T1-weighted image | diffusion weighted image |
| --- | --- | --- |
| Manufacturer | Siemens 3T TrioTim | Siemens 3T TrioTim |
| Repetition time (ms) | 2300 | 9200 |
| Echo time (ms) | 2.98 | 82 |
| Slice Thickness (mm) | 1 | 2.7 |
| Voxel size | 1 x 1 x 1 | 2.73 x 2.73 x 2.73 |
| b-value (s/mm^2^) | - | 1000 |
| directions | - | 44 |
| Phase encoding direction | - | Anterior-Posterior |
|  |  |  |

**Abbreviations:** mm = mulimeters; ms = miliseconds; s = seconds.
